# Increased complications of periacetabular osteotomy in the obese patient is not a contraindication

**DOI:** 10.1093/jhps/hnaf038

**Published:** 2025-08-04

**Authors:** Jordan Boivin, Juliana Overbey, Thomas Ryan

**Affiliations:** Western Michigan Homer Stryker MD School of Medicine, 300 Portage St, Kalamazoo, MI 49007, United States; Western Michigan Homer Stryker MD School of Medicine, 300 Portage St, Kalamazoo, MI 49007, United States; Department of Orthopaedics, Western Michigan Homer Stryker MD School of Medicine, 1000 Oakland Dr, Kalamazoo, MI 49008, United States

## Abstract

Periacetabular osteotomy (PAO) is performed to relieve symptomatic acetabular dysplasia by reorienting the acetabulum to provide adequate femoral head coverage. Due to an increased risk for complications in obese patients, some surgeons consider obesity as a contraindication for surgery. We theorize obese patients will have similar reductions in their pain levels and improvement in radiographic parameters despite an increased risk for postoperative complications. We performed a retrospective review of all patients who underwent a PAO in the last 12 years by the principal investigator. The incidence of complications, change in visual analogue scale (VAS) scores at 6 months follow up, and change in lateral centre edge angles (LCEAs) were then compared between obese patients and non-obese patient cohorts. Forty-eight hips in 41 patients were analysed. Fifteen hips were of obese patients at time of operation and 33 hips were of non-obese patients. The number of complications in the obesity group was statistically significant with a *P* value of .04, while the number of reoperations was not with a *P* value of .11. There was no significant difference in VAS score preoperatively (*P* = .37) and postoperatively (*P* = .26) between patient cohorts. There was no significant difference in LCEA preoperatively (*P* = .35) and postoperatively (*P* = .26). While obese patients are at increased risk for complication, they have a similar reduction in pain compared to the non-obese cohort. PAO in obese patients will provide a significant reduction in patient’s acetabular pain and subsequently improve their quality of life.

## INTRODUCTION

Periacetabular osteotomy (PAO) is a complex procedure performed to relieve symptomatic acetabular dysplasia by reorienting the acetabulum to provide adequate femoral head coverage [1–3]. Developmental dysplasia of the hip is a leading cause for osteoarthritis that can lead to total hip arthroplasty (THA) in young adults [4–6]. This procedure has been shown to have good survivorship with little to no arthritic change for 80% of patients 20 years postoperatively [7–9]. Potential complications of this highly effective procedure include but are not limited to lateral femoral cutaneous nerve (LCFN) damage, heterotopic ossification (HO), nonunion, malunion, hardware failure, hematoma, wound dehiscence, deep vein thrombosis (DVT), and deep infection [1, 4, 8, 10–12]. It is known that there is a large learning curve for this procedure and complications decrease with experience [1, 13, 14]. Many different investigations have demonstrated that obesity is an independent risk factor for complications [1, 10–12, 15].

Previously, body mass index (BMI) >30 has been a reason to deny the surgical option of PAO to patients with acetabular dysplasia due to the increased difficulty and perioperative complications. Despite these reports of high rates of complications in an obese patient population following a PAO, we believe the beneficial outcomes for these patients outweigh the risks of operating in this patient cohort. We hypothesize that long-term outcomes of obese PAO patients are similar to those of our PAO patients with BMI <30. We hope this retrospective review will assuage surgeon concerns of perioperative complications in their obese patient population due to the positive outcomes it provides the patients.

## MATERIALS AND METHODS

Patients who underwent a PAO were retrospectively examined in this single institution, single surgeon study from January 2012 through December 2022. Inclusion criteria consisted of any patient who underwent PAO during this time with at least 6 months follow up. No patients who underwent PAO were excluded. Patient charts were reviewed for age, sex, BMI, comorbidities, complications, and visual analogue scale (VAS) scores. Obesity was defined as a BMI >30 when analysing the data. Preoperative and postoperative radiographic measurements were obtained utilizing the Merge Unity (MergeUnity™ v.11.0, Wisconsin) picture archiving and communication system. Radiographic measurements of the lateral centre edge angle (LCEA) were obtained on weight bearing radiographs pre- and post-operatively. The LCEA was measured by the attending surgeon.

Descriptive and comparative statistics including average, standard deviation, percentage, relative risk, and *t*-tests were calculated where appropriate using Microsoft Excel. The *t*-test was calculated using an unpaired two-tailed *t*-test and Fisher Exact test. Statistical significance was set at *P* < .05.

## RESULTS

A summary of the demographic data is shown in Table 1. Forty-one patients underwent PAO from January 2012 through December 2022. Seven of these patients underwent a PAO bilaterally. Thus, 48 hips in 41 patients were analysed. The average age was 33 ± 11 years in the obese patients and 32 ± 11 years in the non-obese patients. Ten of the 41 (24%) were male. Twenty-five (52%) of the PAOs were performed on the right side. Fifteen PAOs were performed on obese patients of which there were nine complications and four reoperations. Thirty-three PAOs were performed on non-obese patients of which there were 10 complications and 5 reoperations. Sixty per cent of obese patients experienced a complication compared to 30% of non-obese patients. Thirty-three per cent of obese patients experienced reoperation compared to 15% of non-obese patients. Average follow up was 1.5 years. Our results are summarized in Table 2.

**Table 1:** Demographic information of patients undergoing PAO.

	**Number of patients**	**Average (mean ± SD) or percentage (%)**
Age		
Obese	15	33 ± 11 years
Non-obese	33	32 ± 11 years
Gender		
Male	10	21
Female	38	79
Extremity		
Right	25	52
Left	23	48
BMI		
Obese	15	35.2 ± 4.4
Non-obese	33	23.7 ± 3.2

**Table 2:** Numerical data for complication and reoperation rate, LCEA, and VAS score.

	**Obese hips**	**Non-obese hips**	**Statistical analyses (*P* value)**
Total hips	15	33	
Complications	9 (60%)	10 (30.3%)	.04
Required reoperation	5 (33%)	5 (15.1%)	.11
Mean preoperative LCEA	14.4 ± 7.4°	15.1 ± 5.2°	.74
Mean postoperative LCEA	36.4 ± 6.3°	35.3 ± 5.1°	.55
Mean preoperative VAS	5.6 ± 2.0	5.8 ± 2.0	.73
Mean postoperative VAS	1.7 ± 1.9	2.1 ± 2.4	.52

In the obese population, there were two incidences of failure of hardware, one nonunion, two incidences of HO, and three incidences of wound dehiscence. There were no deep infections in the obese patient population. Three patients had a BMI over 40, of which two had a complication. In the non-obese population, there were a total of five incidences of failure of hardware, one case of nonunion, one DVT, and one incidence of HO. There was one case with a deep infection. The number of complications in the obesity group was statistically different with a *P* value of .04.

In the obese patients, reoperations included three superficial irrigation and debridements for wound dehiscence and operative fixation of a nonunion requiring autogenous bone grafting. In non-obese patients, the reoperations included an excision of HO and hardware, a removal of hardware with psoas release, an irrigation and debridement for deep infection, and a nonunion of the ischium requiring fixation and grafting. None of the patients were converted to a THA. The number of reoperations was not statistically different with a *P* value of .11.

The mean of obese patients’ preoperative VAS was 5.6 ± 2.0 and postoperative VAS score 1.7 ± 1.9. Non-obese patients’ preoperative VAS score was 5.8 ± 2.0 and postoperative VAS score 2.1 ± 2.4. There was no significant difference in VAS score preoperatively (*P* = .733) and postoperatively (*P* = .518). On radiographic analysis, obese patients’ preoperative LCEA was 14.4 ± 7.4° and postoperative LCEA 36.4 ± 6.3°. Non-obese patients’ preoperative LCEA was 15.1 ± 5.2° and postoperative LCEA 35.3 ± 5.1°. There was no significant difference in LCEA preoperatively (*P* = .739) and postoperatively (*P* = .545).

## DISCUSSION

PAO’s are an effective procedure to benefit the quality of life of patients with symptomatic acetabular dysplasia. We need to understand which patients would best benefit from the operation. Obesity has been used as a justification to not offer patients a PAO due to the increased difficulty and complication rate [1, 8, 10–12, 14]. Our results showed increased complication and reoperation rates in obese patients undergoing a PAO similar to prior studies [1, 7, 10–12, 14, 15]. The lack of statistical difference in the reoperation rate was likely due to the low sample size, but the trend is consistent with prior literature. Despite these increased rates, VAS score and LCEA were not significantly different between patient groups. The pain reduction was statistically the same when comparing obese patients to non-obese patients. The reorientation of the acetabulum determined by the normalization of the LCEA was statistically the same despite the increased difficulty of the procedure. This is similar to what was reported by Novais et al., that obese patients undergoing PAO have similar outcomes despite the increased complication and reoperation rates [1]. Our centre is not a large academic centre that performs this procedure in large volumes. Our similar complication rates and outcome measures with prior literature in large academic centres demonstrate that this procedure can be performed in a smaller centre with comparable results.

This study has limitations. First, after thorough chart review, we did not have the required data pre- and post-operatively to calculate a verified hip outcome score for our patient population. We had to rely on radiographic measurements to determine a radiographic outcome and decreased VAS scores to infer a clinical benefit. Second, this was a retrospective chart analysis. This lends to potential missed complications that were not documented in the chart. Third, a single surgeon performed these procedures at a private institution. With the steep learning curve, this could have resulted in more complications in the earlier procedures. This could limit the generalizability of these results to larger institutions performing more of these procedures. Lastly, the amount of follow up varies depending on when the procedure was performed and could result in missing future long-term complications.

Based on our results, we believe that obesity alone should not be used to deny a patient a PAO. With adequate preoperative discussion, we can set realistic expectations of good outcomes despite these complications. The author does not have a cut off BMI for the procedure. The author does encourage weight loss prior to the procedure, however, does not delay surgical intervention due to BMI as long as a thorough preoperative discussion about the increased risks was had and the patient agrees to proceed with the procedure. Additional time, equipment needs, and support services did not play a role in whether or not these patients were indicated for surgery. Our goal is to reduce acetabular pain and prolong or even eliminate the future need for a THA.

Our findings indicate that the reduction in pain was not significantly different between obese and non-obese patients despite the increased number of complications. Both patient populations were able to achieve normalized postoperative LCEAs. These findings lead us to conclude that PAO in obese patients will provide a significant reduction in patients’ acetabular pain and subsequently improve their quality of life. Further studies in a larger patient population would provide increased power to further investigate the statistical and clinical significance of a patient’s obesity on their outcomes from PAO.

## Data Availability

The data underlying this article will be shared on reasonable request to the corresponding author.
